# Amelioration of Alzheimer’s Disease by Gut-Pancreas-Liver-Brain Interaction in an App Knock-In Mouse Model

**DOI:** 10.3390/life12010034

**Published:** 2021-12-27

**Authors:** Mayumi Minamisawa, Yuma Sato, Eitarou Ishiguro, Tetsuyuki Taniai, Taiichi Sakamoto, Gota Kawai, Takashi Saito, Takaomi C. Saido

**Affiliations:** 1 Department of Life Science, Chiba Institute of Technology, Graduate School of Advanced Engineering, Chiba 275-0016, Japan; s16c2063nz@s.chibakoudai.jp (Y.S.); taiichi.sakamoto@p.chibakoudai.jp (T.S.); gota.kawai@p.chibakoudai.jp (G.K.); 2 Education Center, Faculty of Advanced Engineering, Chiba Institute of Technology, Chiba 275-0023, Japan; drtaniai@mx3.ttcn.ne.jp; 3 i-on Co., Ltd., Chiba 263-0032, Japan; ishiguro@i-ongroup.com; 4 RIKEN Center for Brain Science, Laboratory for Proteolytic Neuroscience, Saitama 351-0198, Japan; saito-t@med.nagoya-cu.ac.jp (T.S.); takaomi.saido@riken.jp (T.C.S.); 5 Department of Neurocognitive Science, Institute of Brain Science, Nagoya City University Graduate School of Medical Sciences, Nagoya 467-8601, Japan

**Keywords:** Alzheimer’s disease, L-arginine, limonoids, polyamine, intestinal microbiota, next-generation sequencing, bacterial translocation, pancreatitis, metabolome analysis

## Abstract

In this study, we observed disease progression, changes in the gut microbiota, and interactions among the brain, liver, pancreas, and intestine in a mouse model of Alzheimer’s disease (AD), in addition to attempting to inhibit disease progression through the dietary supplementation of L-arginine and limonoids. Wild-type mice (WC) and AD mice were fed a normal diet (AC), a diet supplemented with L-arginine and limonoids (ALA), or a diet containing only limonoids (AL) for 12–64 weeks. The normal diet-fed WC and AC mice showed a decrease in the diversity of the gut microbiota, with an increase in the Firmicutes/Bacteroidetes ratio, and bacterial translocation. Considerable bacterial translocation to the pancreas and intense inflammation of the pancreas, liver, brain, and intestinal tissues were observed in the AC mice from alterations in the gut microbiota. The ALA diet or AL diet-fed mice showed increased diversity of the bacterial flora and suppressed oxidative stress and inflammatory responses in hepatocytes and pancreatic cells, bacterial translocation, and neurodegeneration of the brain. These findings suggest that L-arginine and limonoids help in maintaining the homeostasis of the gut microbiota, pancreas, liver, brain, and gut in AD mice.

## 1. Introduction

The 21st century is the century of the brain and mind (Joint statement of the G7/G8 summit by the G-Science Councils), with a need for understanding the brain, protecting it against disease, and developing global brain-related resources [1,2]. Brain diseases are a global challenge to individual health, well-being, economic productivity, and intellectual capital [3,4].

There are several types of dementia with various causes; however, Alzheimer’s disease (AD) is the most common; approximately 50 million people globally have AD. In Ja-pan, there are more than 7 million people have dementia, of which approximately 5 million have AD [5].

AD is a specific neurodegenerative disease in which progressive nerve inflammation and damage lead to dementia. One of the suspected factors contributing to the development of AD is the amyloid cascade hypothesis [6,7], which proposes that the accumulation of amyloid-β (Aβ) and tau protein in the foci of neuronal destruction or near-death in the brain results in the disruption of the homeostatic functions of the brain, resulting in neuronal toxicity. Several hypotheses have been proposed regarding the etiology of AD [8,9]. The neuropathology, common to all hypotheses, comprises senile plaques, neurofibrillary changes, and elevated levels of neuroinflammation. Various treatments have been investigated, including the development of drugs that inhibit the production of Aβ₋tau; targeting the production, aggregation, and deposition of Aβ [10,11] and production of reactive oxygen species in neurons [12]; the utilization of apoptosis and development of inhibitors of inflammation; and stem cell-based therapy [13,14]. Unfortunately, no treatment exists to reverse the progression of AD. There is a strong belief that AD is a brain disease. However, the pathogenesis of AD is also known to be caused by other systems, genetic factors, cardiovascular health, type 2 diabetes, metabolic syndrome, smoking, traumatic brain injury, and immune dysfunction [15,16]. The interaction of multiple genetic risk factors and environmental factors contributes to the onset and progression of AD, and there is an urgent need for treatments. Although administering medication to subjects at a high risk of developing the disease for an extended period is desirable before the onset of the disease, the side effects, duration of administration, and cost remain problematic; therefore, preventive medication that is inexpensive and safe for long-term prevention is essential.

Although elucidation of the underlying mechanism currently lingers, we consider degeneration of the nervous system to be rooted in oxidative stress and inflammation, given that diet is well known for moderating these phenomena. For instance, there are reports on brain–gut correlations, wherein signals in the gut caused by food are transmitted directly to the brain via the vagus and sensory nerves [17].

The relationship between the gut microbiota and disease is well documented in conditions such as allergies and asthma [18], inflammatory bowel disease [19], obesity-related diseases [20], and systemic inflammation. Changes in microbiota composition are believed to mediate neurodevelopmental or neurodegenerative conditions [21,22]. Extensive evidence is available on the association of the behavior of the gut microbiota with many of the critical homeostatic processes that help maintain a healthy human immune system. We explored the pathogenesis of a mouse model of Sandhoff disease (SD), a neurological condition caused by thalamic neurodegeneration for which no reliable treatment is available and found dysbiosis in the gut microbiota of mice with SD [23]. We reported about a novel therapy which dietary supplementation using the yuzu seed extract limonoid and spermine controlled the diversity of gut microbiota in mice. The metabolites produced by the gut microbiota and immune response were found to suppress neurodegeneration in the thalamus and extend lifespan [23]. Although the mechanisms underlying increased intestinal permeability in dysbiosis remain elusive, one important factor is that the limonoid diet inhibited the increased intestinal permeability in SD mice. Dysbiosis is also believed to occur in the gut of patients at the onset of AD [24].

In this study, we aimed to examine the changes in the gut microbiota with the progression of AD and to simultaneously identify a mechanism that could lead to the development of inexpensive preventive drugs for AD via the gut microbiota. Based on the results of our previously published studies [23,25], we selected a diet of L-arginine and yuzu seed-derived limonoids to maintain homeostasis in AD mice via brain–gut interactions and to inhibit or alleviate the progression of the disease. Microglia in the brain reportedly divide and change and begin to consume large amounts of arginine during the early stages of the pathogenesis of neurodegenerative diseases [26]. L-arginine has already been administered as a drug for the treatment of congenital urea cycle abnormalities and mitochondrial encephalomyopathy. Furthermore, it can penetrate the blood–brain barrier (BBB) and be taken up into the brain.

No toxicity due to overaccumulation in the host from the exogenous administration of L-arginine has been reported, and the administration of L-arginine to AD mice is expected to help maintain mitochondrial physiological functions. L-arginine is metabolized to NO and L-citrulline by the L-arginine–nitric oxide pathway. L-arginine exerts its influence on the cardiovascular system by appropriately increasing the production of NO in the nitric oxide synthase pathway [27], i.e., a deficiency of L-arginine deficiency in the vascular endothelium may cause NO deficiency, an essential molecule in the prevention of atherosclerosis [28]. In the neuropathology of AD, many relationships with atherosclerosis have been reported [29,30]. Hypercholesterolemia is a well-known risk factor for atherosclerosis and can cause endothelial dysfunction and vascular cell damage [31], as well as impaired L-arginine metabolism [32]. Therefore, the availability of exogenous L-arginine may be important to suppress the pathogenesis of AD.

Furthermore, L-arginine is involved in the metabolic pathway of arginase–polyamine synthesis. We suggested the effect of the interaction between a limonoid diet and polyamines as one of the mechanisms for the prevention of intestinal microbiota disturbance in the SD mice model of central nervous system disease [23].

Thus, polyamines, which are metabolized and produced from L-arginine, contribute significantly to the gut microbiota and host health, especially with the maintenance of the intestinal mucosal barrier [23,33] and promotion of autophagy [34]. Furthermore, polyamines can reportedly inhibit the enhanced phosphorylation and intracellular accumulation of tau, a microtubule aggregation protein in AD [35]. Therefore, exogenous L-arginine and limonoids may be an important molecular combination for use in the treatment of AD. In the present study, we attempted to control the balance between the pathways of nitric oxide and polyamine synthesis pathways by administering L-arginine, as well as the dietary supplementation of limonoids to the AD mice model to maintain the gut microbiota involved in host homeostasis and to inhibit disease progression. Furthermore, we examined the behavior of the gut microbiota, cytokines, chemokines, and signaling molecules during disease progression in the AD mice model and examined the interactions among the brain, liver, pancreas, gut, and gut microbiota.

## 2. Materials and Methods

### 2.1. Materials

As reported in a previous study, the limonoids used in the experiments were extracted and purified from yuzu seeds [36]. Briefly, the limonoid mixture extracted and purified from yuzu seeds purchased from Kyoto Mizuo (Japan) consisted of deacetylnomilin (105 mg g^−1^), limonin (95 mg g^−1^), nomilin (115 mg g^−1^), and obacunone (17 mg g^−1^) per gram of dried yuzu seeds. In addition to the limonoid component, the yuzu seeds were also a good source of vitamin C.

Compositional determination of limonoid components and yuzu seed oil was conducted using high-performance liquid chromatography–mass spectrometry and gas chromatography analyzers from Shimadzu Systems (Kyoto, Japan) [25]. The standard analytical reagents or ultrapure reagents used in the analysis were purchased from Wako Pure Chemical Company (Osaka, Japan). To feed mice with limonoids, 500 g of lipophilic limonoids extracted and purified from yuzu seeds were commissioned to Nippon Clare Company (Tokyo, Japan), which prepared 10 kg of γ-ray sterilized feed after mixing and molding the lipophilic limonoids into commercial mouse food.

### 2.2. Mice

All mice used in this study were bred and raised under standard non-sterile conditions. All mice, including wild-type (WT) mice which are essential from the point of view of AD research, were provided by the RIKEN BRC through the National Bio-Resource Project of the MEXT/AMED, Japan. The knock-in mice were generated using C57B-derived ES cells, in which a genetic mutation was inserted into the amyloid-β region of the mouse App gene, by Dr. Takashi Saito and Dr. Takaomi Saido at the RIKEN Brain Science Institute (Saitama, Japan) [37]. A Swedish mutation alone (App<NL> knock-in, RBRC06342), a double mutation with an Iberian mutation (App<NL-F> knock-in, RBRC06343), and a triple mutation with an Arctic mutation (App<NL-G-F> knock-in, RBRC06344) were introduced. App<NL> knock-in mice were used as negative controls. App<NL-G-F> homozygous mice showed the development of amyloid plaques in the brain from 2 months of age. App<NL-G-F> knock-in mice showed behavioral abnormalities in the Y-maze from 6 months of age.

### 2.3. Investigation of the Effects of L-Arginine and Limonoid Administration on Mouse Models

In the present study, C57BL/6-App<tm3(NL-G-F)Tcs> AD mice were fed only a commercial diet (Nippon Clea Co., Ltd., Tokyo, Japan) as part of the AD control (AC) group. Mice fed with the diet containing limonoids and 0.17–0.20 mg of L-arginine dissolved in 200 mL of drinking water were designated as AD mice fed limonoids + L-arginine (ALA), and mice fed only with the diet containing limonoid were designated as AD mice fed limonoids only (AL). The negative control App<NL> knock-in mice, as the wild type, were designated as WC and fed only a commercially available diet specifically designed for mice.

Therefore, the AD mice (*n* = 16; males only) consisted of the following three groups: AC group, *n* = 4; ALA group, *n* = 4; and AL group, *n* = 4. The WC group consisted of five male mice only.

Dietary restriction was started from 8 weeks of age until approximately 53–64 weeks of age, when the animals died, and changes in body weight were measured. At 53 weeks, a humane endpoint was applied, which was when the AC mice first became moribund and could no longer attain an acceptable health status. For anesthesia, 25 mL of a triple mixture of anesthetics [38] (1.875 mL (mouse required dose; 0.75 mg kg^−1^) Domitor (Nippon Zenyaku Kogyo Co., Ltd., Koriyama, Japan), 2 mL (mouse required dose; 4 mg kg^−1^) midazolam “SANDOZ” (Sandoz Co., Ltd., Tokyo, Japan), 2.5 mL (mouse required dose; 5 mg kg^−1^) Vetorphale (Meiji Pharma Co., Ltd., Tokyo, Japan) in 18.625 mL of saline) was prepared as per the preparation ratio, and 0.1 mL per 10 g of body weight of mice was injected intraperitoneally.

The mean daily intake (g) ± standard deviation (SD) of limonoids and L-arginine in the ALA group from 12 to 64 weeks was 5.42 g ± 0.710 g mouse kg^−1^ day^−1^ and 0.15 g ± 0.031 mouse kg^−1^ day^−1^, respectively, whereas that of limonoids in the AL group was 5.37 g ± 0.797 mouse kg^−1^ day^−1^.

### 2.4. Fecal Samples and DNA Extraction

Fecal samples (50–100 mg) were collected from mice until 12–64 weeks of age in 2-mL tubes (Eppendorf, Hamburg, Germany) and processed to extract DNA. The QIAcube robotic workstation system for automated purification of DNA with the QIAGEN spin-column Kit and the QIAamp PowerFecal Pro DNA Kit (Qiagen, Germany) were used; DNA extraction was performed in accordance with the manufacturer’s instructions (Qiagen, Germany). DNA was eluted in 100 μL of elution buffer included in the kit. For individual fecal samples, DNA was extracted with samples collected each week in triplicates for each sample; thus, DNA samples for further analyses (each sample × triplicate). The quantity of DNA was measured using an Invitrogen Qubit 4 fluorometer (Thermo Fisher Scientific, Tokyo, Japan) and remaining DNA was stored at −20 °C until further use.

### 2.5. Mouse Tissue Samples and DNA Extraction

The tissue samples (brain, pancreas, kidney, spinal cord, liver, large intestine, small intestine, spleen, heart, white cells, and stomach; approximately 25 mg each) were collected in 2 mL tubes (Eppendorf, Germany) until the mice were 53 or 64 weeks of age and used for DNA extraction experiments. Herein, a QIAcube robotic workstation system for automated DNA purification using the QIAGEN spin-column Kit (DNeasy Blood & Tissue Kit; Qiagen, Germany) was used. DNA extraction was performed following the manufacturer’s instructions, using a QIAcube system (Qiagen, Germany). DNA was eluted in 200 μL of elution buffer included in the kit. From the individual tissue samples, DNA was extracted thrice from each tissue sample and the DNA samples were kept for further analyses (three samples from each group). The quantity of DNA was measured using an Invitrogen Qubit 4 fluorometer (Thermo Fisher Scientific, Japan) and the remaining DNA was stored at −20 °C until further use.

### 2.6. Gut Microbiota Analysis: 16SrRNA Amplicon Sequencing Library Preparation

The Illumina protocol “16S Metagenomic Sequencing Library Preparation, preparing 16S Ribosomal RNA (v4), Gene Amplicons for the Illumina Miseq/MiniSeq System” was used. The variable V4 region of 16S rRNA was amplified from bacterial DNA extracted from feces. The most promising bacterial primer pair for polymerase chain reaction (PCR) amplification was the 16S amplicon (forward primer (5′-TCGTCGCCAGCCGTCAGATGTGTATAGAGACAGGTGYCAGCMGCCGCGG TAA-3′) and reverse primer (5′-GTCTCGTGCCTCGGAGATGTGTATAAGAGACAGGGA CTACHVGGGTWTCTAAT-3′)). The primers were synthesized using the Hokkaido System Science Co. (Hokkaido, Japan), and an Illumina (San Diego, CA, USA) adapter overhanging a nucleotide sequence was added to the 5′ end of both primers. The prepared 16s ribosomal RNA gene amplicons for the illumina MiniSeq system used in the NGS were executed as reported in a previous study [23]. The DNA concentration of the prepared libraries was determined by Qubit (Thermo Fisher, Waltham, MA, USA) to confirm the concentration by the fluorescence quantification method, which allows specific detection of duplex DNA and conversion into nM units, as required. Based on this calculation, all libraries were pooled in equimolar amounts, denatured, and diluted to 1.5 pM before loading into the MiniSeq flow cell and sequencing on the Illumina MiniSeq platform (Illumina, San Diego, CA, USA).

### 2.7. Microbiota Analysis of Sequencing Data

The raw sequencing files from both amplicon primers were processed using the QIIME™ 2 [39] microbiome analysis pipeline (https://docs.qiime2.org (accessed on 9 March 2021), ver. 2021.2). The method and plugins (https://github.com/qiime2/ (accessed on 9 March 2021)) were used. The “tools import” (https://docs.qiime2.org/2021.2/tutorials/importing/ (accessed on 9 March 2021)) method was used to import the raw sequencing files as the format paired-end sequence with quality score by Phred [33] and to create the “artifact” file (i.e., the qiime2 data format required for subsequent analyses). The “deblur denoise-16S” (https://docs.qiime2.org/2021.2/plugins/available/deblur/ (accessed on 9 March 2021)) plugin was used for quality control of Illumina data using the Deblur workflow (https://github.com/biocore/deblur (accessed on 9 March 2021)) and to make the feature table and representative sequence (i.e., amplicon sequence variants (ASVs)) data. The “feature-classifier classify-sklearn” (https://docs.qiime2.org/2021.2/plugins/available/feature-classifier/classify-sklearn/ (accessed on 9 March 2021)) plugin was used for classifying these ASVs into biological taxonomy using the SILVA SSU database release 138 (https://www.arb-silva.de/documentation/release-138/ (accessed on 5 May 2021)) [40]. The taxon summary produced bar plots (https://docs.qiime2.org/2021.2/plugins/available/taxa/ (accessed on 9 March 2021)) according to the sample groupings. The heatmap representation of the feature table was generated by the “feature-table heatmap” plugin (https://docs.qiime2.org/2021.2/plugins/available/feature-table/heatmap/ (accessed on 9 March 2021)). Additionally, linear discriminant analysis effect size (LEfSe) (http://huttenhower.sph.harvard.edu/galaxy/ (accessed on 15 August 2021)) was performed to investigate the gut bacterial biomarkers that characterized each group of mice [41].

### 2.8. Histopathological Analysis of Mice Tissues

Histopathological findings of the brain, pancreas, spinal cord, liver, small intestine, and other tissues of the AC (53 weeks of age), WC (64 weeks of age), and ALA and AL (64 weeks of age) mice whose survival was prolonged by a diet of limonoids + L-arginine and limonoids only, were compared. In addition, histopathological analysis of the brain, pancreas, spinal cord, liver, and small intestine was performed. Each formalin-fixed tissue was embedded in paraffin, cut into 4 μm sections, and stained with hematoxylin and eosin (H&E). A bright-field slide scanner was used to capture the entire image of the H&E-stained specimen using a 20× objective lens setting (Genetic Lab Corp. Ltd., Sapporo, Japan, http://www.gene-lab.com (accessed on 17 August 2021)).

Unstained brain specimens were prepared for immunofluorescence staining of glial fibrillary acidic protein (GFAP), ionized calcium-binding adapter protein 1 (Iba1), and triple fluorescence staining of amyloid plaques using Amyloid-Glo. For GFAP staining, chicken-derived anti-GFAP antibody (Abcam plc.) and Goat anti-Chicken IgY (H + L) (Thermo Fisher Scientific Inc., Tokyo, Japan) were used as the primary and secondary antibodies, respectively, conjugated with Alexa Fluor 488 (Thermo Fisher Scientific Inc., Tokyo, Japan).

For Iba1 staining, rabbit-derived anti-Iba1 antibody (Abcam plc.) was used as the primary antibody, and Goat anti-Rabbit IgG (H + L) Cross-Adsorbed secondary antibody (Thermo Fisher Scientific Inc., Waltham, MA, USA) was used as the secondary antibody; they were stained with Alexa Fluor 568 (Thermo Fisher Scientific Inc., Waltham, MA, USA). In addition, proLong Gold Antifade Reagent with DAPI (Thermo Fisher Scientific Inc., Waltham, MA, USA) was used for inclusion.

For amyloid staining, Amylo-Glo RTD Amyloid Plaque Stain Reagent was used and sealed with proLong Gold Antifade Reagent with DAPI. The prepared, stained specimens were photographed using a confocal image cytometer with a 40× objective lens (Genetic Lab Corp. Ltd. Sapporo, Japan, http://www.gene-lab.com (accessed on 20 August 2021)). Images were taken independently for each specimen, and lastly, superimposed images of GFAP (green), Iba-1 (red), and amyloid (blue) were prepared.

### 2.9. Measurement of Cytokine/Chemokine Concentration in Mouse Blood

The concentrations of cytokines/chemokines in the blood of AC, ALA, AL, and WC mice were measured by the Multiplex Suspension Array System (Luminex^®^ 100/200TM System; Luminex Corp., Austin, TX, USA) using the MILLIPLEX^®^ MAP Mouse Cytokine/Chemokine Magnetic Bead Panel (Merck Millipore Corp., Burlington, MA, USA). Blood samples were collected from AC, ALA, AL, and WC mice at 30 weeks and 53 (AC only) or 64 weeks and centrifuged at 2300× *g*, for 15 min at 4 °C to collect serum. Levels of Interleukin (3, 5, 7, 9, 10, 13, 15, and 17), tumor necrosis factor (TNF)-α, granulocyte colony-stimulating factor (G-CSF), eotaxin, macrophage inflammatory protein (MIP)-1α, MIP-1β, blood levels of LIX, KC, and RANTES (regulated on activation, normal T-cell expressed and secreted) were measured.

### 2.10. Metabolome Analysis of Mouse Liver by Capillary Electrophoresis–Time-of-Flight Mass Spectrometry

Metabolome analysis of mouse liver was performed using a capillary electrophoresis–time-of-flight mass spectrometry (CE-TOFMS) system (Agilent CE-TOFMS System, Agilent Technologies, Tokyo, Japan). The cationic and anionic metabolites were analyzed simultaneously by electrospray ionization (ESI) (positive and negative) MS method using a fused silica capillary with an i.d. of 50 µm × 80 cm for *m*/*z* values of 50–1000 (Agilent Technologies, Japan). The metabolites were analyzed simultaneously by the Human Metabolome Technologies, Inc., (HMT Inc., Yamagata, Japan). A tissue sample of approximately 11.7–12.5 mg was collected from the same region of the liver of AC (53 weeks of age), ALA, and AL mice (64 weeks of age) (*n* = 4, each). In total, 46.9–49.0 mg was prepared by mixing four liver tissues for each mouse model. To the collected tissue samples, 750 µL of 50% acetonitrile solution (*v*/*v*) (standard internal concentration: 20 µM) was added, and the samples were crushed (1500 rpm for 120 s × 3 times) using a crusher under cooling. To this, 750 µL of 50% acetonitrile solution (*v*/*v*) was added. After tissue disruption, centrifugation (2300× *g*, 4 °C, 5 min) was performed. After centrifugation, 400 µL of the upper layer was transferred to an ultrafiltration tube, then centrifuged (9100× *g*, 4 °C, 120 min) and subjected to ultrafiltration. The filtrate was dried and dissolved again in 50 µL of Milli-Q water for measurement.

### 2.11. Metabolome Analysis of Mouse Feces by Liquid Chromatography/Time-of-Flight Mass Spectrometer

Metabolome analysis was performed using liquid chromatography/time-of-flight mass spectrometer (LC-TOFMS) measurement (HMT Inc.). Feces samples from AC, ALA, AL, and WC mice (64 weeks of age) (*n* = 4, each) from each mouse model were collected in the amount into microtubes (Microspitz 1.5 mL, Greiner, Frickenhausen, Germany) so that the total amount ranged from 30.0 mg to 50.0 mg, respectively.

Ultrapure water containing internal standard (20 μM: provided by HMT Inc.) was added to the fecal samples at a ratio of 1:9 by weight. The fecal samples were shaken with a shaking apparatus (Scientific Industries Inc., Bohemia, NY, USA) until they were completely dissolved and centrifuged (2300× *g*, 4 °C, 5 min). After centrifugation, 350 µL of the upper layer was transferred to an ultrafiltration tube. The sample was centrifuged (9100× *g*, 4 °C, 120 min), subjected to ultrafiltration, and 100 µL of the filtrate was dried and dissolved in 400 µL of 50% isopropanol solution (*v*/*v*) to prepare the measurement sample. Liquid chromatographic analysis was performed under the folllowing conditions: ODS column, 2 mm × 50 mm, 2 μm; column temperature, 40 °C; mobile phase A, H_2_O/0.1% HCOOH, mobile phase B, isopropanol:acetonitrile:H_2_O (65:30:5)/0.1% HCOOH, 2 mM HCOONH_4_; flow rate, 0.3 mL/min; run time, 20 min; post time, 7.5 min; gradient condition: 0–0.5 min, 1% B; 0.5–13.5 min, 1–100% B, 13.5–20 min, B. MS was performed by the Agilent LC/MSD TOF (Agilent Technologies) using the ESI (positive and negative) MS method to simultaneously analyze cationic and anionic metabolites from *m*/*z* 100–1700.

### 2.12. Processing and Analysis of Metabolome Analysis Data

Peaks detected by CE-TOFMS and LC-TOFMS were automatically extracted using the automated integration software MasterHands ver. 2.17.1.11 (Keio University, Japan) for peaks with a signal-to-noise (S/N) ratio of 3 or higher, and the mass-to-charge ratio (*m*/*z*), peak area values, and swimming time (MT) were obtained. Then, putative metabolites were assigned from HMT’s standard library based on the determined *m/z* and migration time. Absolute quantification of the target metabolites was simultaneously performed by normalizing the peak area of each metabolite to the area of the internal standard. Principal component analysis, hierarchical clustering analysis, and heatmap notation were performed using a statistical analysis software (developed by HMT). In both analyses, the data were preprocessed by peak-wise standardization (μ = 0, σ = 1). The metabolic pathways were designed based on the enzymes identified in humans.

In the metabolomic analysis of the mouse liver, a candidate compound was assigned to 331 (cation 196, anion 135) peaks from the *m*/*z* and MT values of substances registered in the HMT metabolite Known–Unknown library based on metabolomic analysis by CE-TOFMS.

In contrast, in metabolomic analysis using mouse feces by LC-TOFMS, candidate compounds were assigned to 191 (105 positives, 86 negatives) peaks based on the *m*/*z* and RT values of the substances registered in the HMT metabolite library, and the relative area value ratios were calculated for all the candidate compound peaks.

### 2.13. Statistical Analysis

We used a Kruskal–Wallis test for multiple pairwise comparisons to evaluate significant differences in the relative abundance of bacterial populations. In addition, CSV data on bacterial taxonomy was exported from QIIME2 (f taxa-bar-plots.qzv) for comparison with other experimental parameters.

Continuous parametric data were compared using a one-way analysis of variance, which was followed by Tukey’s post hoc analysis. Unpaired observations were analyzed using a one-sided Student’s *t*-test. The significance threshold was set at *p* < 0.05. Data are expressed as mean ± SD. The correlation between the variables of the measured data was analyzed using the Pearson product-moment correlation coefficient.

Cytokine and chemokine levels in mouse blood were expressed as the mean ± SD, and one-way analysis of variance and *t*-tests were performed for analysis between groups. The threshold for significant differences was set at *p* < 0.05.

## 3. Results

### 3.1. Changes in the Gut Microbiota of Mice Treated with Limonoids and L-Arginine in a Mouse Model of AD

**Figure 1:**
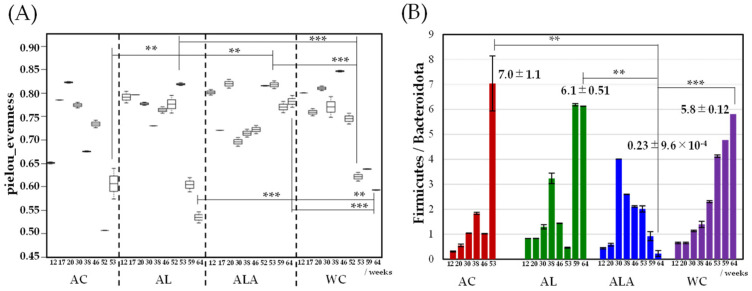
Alpha diversity of genus taxonomic categories identified by genomic analysis of 16S rRNA in feces of Alzheimer’s disease (AD) mice aged 12–64 weeks (AC mice fed a normal diet, AL mice fed a diet containing only limonoids, and ALA fed a diet supplemented with L-arginine and limonoids; *n* = 4/each group, or WC mice fed a normal diet; *n* = 5) (**A**), and the relative abundance Firmicutes/Bacteroidota ratio (**B**). Values represent mean ± S.D. AC mice were treated at 12–53 weeks of age. Data in (**A**) are plotted in comparison with the AD controls (AC) for clarity; * *p* ≤ 0.05, ** *p* ≤ 0.01, *** *p* ≤ 0.001.

As shown in Figure 1A, the alpha diversity of the intestinal microbiota was the highest at 20 weeks for AC, 53 weeks for AL, and 46 weeks for WC, suggesting that ALA supplementation maintained a rich microbiota until the final 64 weeks. In the AC group, the diversity of the bacterial flora significantly decreased 52 weeks onward, the week before the first mouse died, suggesting the occurrence of dysbiosis [41], which is a breakdown in the maintenance of intestinal microflora homeostasis. Conversely, the bacterial flora in the AL and ALA groups-maintained diversity at 53 weeks and was significantly different from that in the AC group. However, the bacterial flora diversity in WC decreased from 53 weeks and that in the AL group decreased from 59 weeks.

In Figure 1B, the gut microbiota in mouse feces was dominated by the phyla Bacteroidetes and Firmicutes, and the temporal change in the abundance of Firmicutes and Bacteroidetes was different in AC, AL, ALA, and WC mice. In AC, AL, and WC mice, the Firmicutes/Bacteroidetes ratio increased rapidly from 53 to 59 weeks. In the AC (53 weeks), AL, and WC (64 weeks) groups, in which the diversity of bacterial flora decreased significantly (Figure 1A), the Firmicutes/Bacteroidetes ratio also decreased significantly (AC, 7.0 ± 1.1; AL. 6.1 ± 0.51; WC, 5.8 ± 0.12). Particularly, AL mice, which were fed limonoids, had a high proportion of Bacteroidetes flora until week 53; in the feces of ALA mice fed L-arginine + limonoids, the Firmicutes/Bacteroidetes ratio was 0.23 even at 64 weeks. In the feces of ALA mice fed L-arginine + limonoids, the Firmicutes/Bacteroidetes ratio was 0.23 ± 9.6 × 10^−4^; *p* < 0.01) even at 64 weeks, and the proportion of Bacteroidetes remained high.

**Figure 2:**
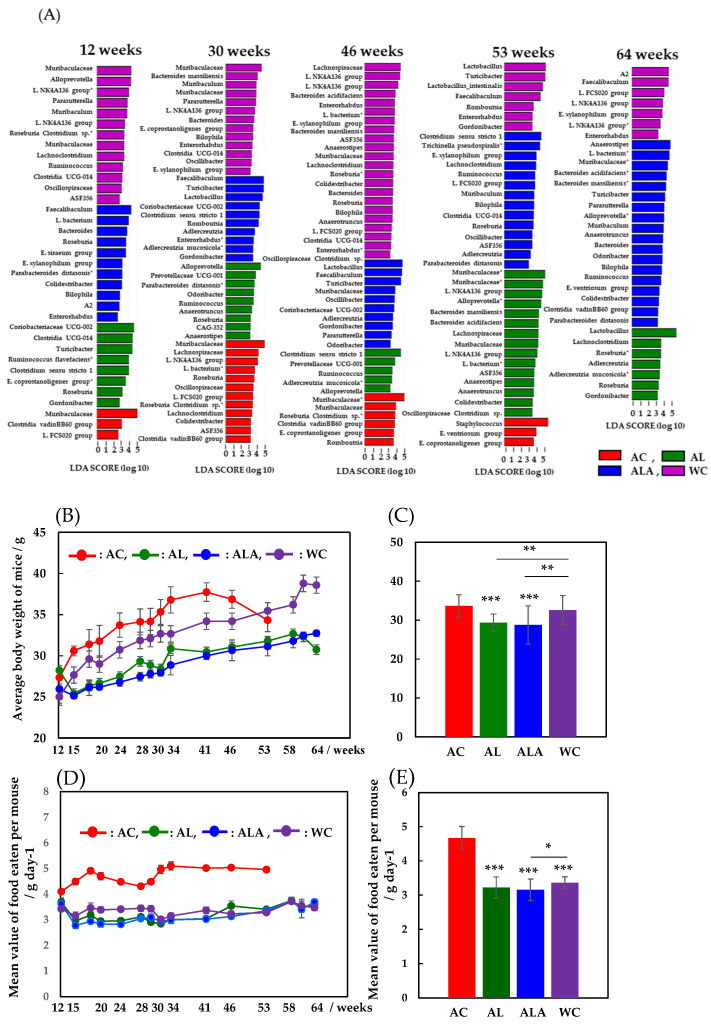
Characterization of time dependence aged 12–64 weeks in microbiomes for Alzheimer’s disease (AD) mice and wild-type mice by LEfSe analysis (**A**). AD mice were fed a diet containing limonoids alone (AL), L-arginine + limonoids (ALA), or normal diet alone (AC). Wild-type mice were fed normal diet alone (WC). Histogram of the LDA scores ((log10) > 2, *p* < 0.05) computed for features with differential abundance in AD mice (AC, AL, ALA mice; *n* = 4/each group) and healthy wild-type mice (WC; *n* = 5). AC mice were treated at 12–53 weeks of age. Relative abundance ratio of microbiota was identified on genus taxonomic categories (* bacteria with species taxonomic categories). L.: *Lachnospiraceae*, E.: *Eubacterium*. Time-dependent changes in mean body weight of AC, AL, ALA, and WC mice from 12 to 53 or 12 to 64 weeks (**B**). Average body weights of AC, AL, ALA, and WC mice up to 53 or 64 weeks (**C**). Time-dependent changes in mean value of food eaten per AC, AL, ALA, and WC mouse in g day^−1^ (**D**). Mean value of food eaten per AC, AL, ALA, and WC mouse/g day^−1^ (**E**). AC, ALA, AL mice; *n* = 4/each group, and WC mice; *n* = 5. Values represent the mean ± SD. Data in (**C**,**E**) are plotted in comparison with the AD controls (AC) for clarity; * *p* ≤ 0.05, ** *p* ≤ 0.01, *** *p* ≤ 0.001.

Figure 2A shows a comparison of the time dependence between 12 and 64 weeks at the genus- or species-levels of bacterial flora in mouse feces (bacteria with distinct species are also shown at the species level) using LEfSe analysis, which is characterized by an analysis of variance that considers individual differences. The higher the linear discriminant analysis (LDA) score, the more it is suggestive of bacterial biomarkers that are characteristic of the gut microbiota of AC, AL, ALA, and WC mice [42].

Comparing the gut microbiota of AC and WC mice in the last week of survival, we found that the intestinal microbiota of 53-week-old AC mice was dominated by *Staphylococcus* (*Firmicutes-Bacilli-Staphylococcales-Staphylococcaceae*), which is found to be expressed in fatal infections [43]. Furthermore, *Eubacterium ventriosum* group (*Firmicutes-Clostridia-Lachnospirales-Lachnospiraceae*) and *Eubacterium coprostanoligenes* group (*Firmicutes-Clostridia-Oscillospirales*, *E. coprostanoligenes* group) [44], which are involved in reducing bile acids and cholesterol, were present. Thus, the gut microbiota of the 53-week-old AC mice exhibited dysbiosis, consisting mainly of anaerobic Gram-positive bacteria belonging to the phylum *Firmicutes*.

The gut microbiota of WC mice, whose diversity decreased dramatically from week 53, was dominated by *Turicibacter*, a bacterium that produces short-chain fatty acids (SCFAs), such as butyric acid [45]; *Faecalibaculum*, a well-known antitumor bacterium [46]; *Lactobacillus*, which promotes intestinal health and is widely used in the treatment of irritable bowel syndrome and ulcerative colitis [47]; and Romboutsia, which is important for maintaining the intestinal mucosa [48]. In 64-week-old WC mice, the gut microbiota presented decreased Bacteroidetes, but *Faecalibaculum* [46] and *Lachnospiraceae* (L. FCS020, L. NK4A136) [48,49], which increase in number with age in healthy hosts, remained constant. The abundance of *Lachnospiraceae* also increases in the intestinal lumen of subjects with various diseases, but this group has also been reported to be capable of producing metabolites that are beneficial to the host [49].

These bacteria belong to the Clostridium cluster XIVa of the phylum Firmicutes. The composition of the gut microbiota was found to be completely different in WC with reduced diversity at 53 weeks and in AC in a state of dysbiosis. From 12 weeks, the diversity of the intestinal microflora of AC mice was found to be low, and the bacterial flora of both WC and AC were in a state of dysbiosis, wherein diversity was reduced at week 53 and was completely different. These bacteria belong to the Clostridium cluster XIVa of the Firmicutes phylum.

In contrast, unlike AC and WC, which showed a decrease in bacterial flora diversity at 53 weeks, gut microbiota in 53-week-old AL mice fed a diet of limonoids showed the highest diversity between 12 and 64 weeks. Bacteria belonging to the *Bacteroidales* order of *Muribaculaceae*, *Alloprevotella*, *Bacteroides massiliensis*, and *Bacteroides acidifaciens* were the most predominant in the gut microbiota of AL mice. Bacterial species belonging to *Bacteroidales* are mucolytic bacteria (*mucinolytic* bacteria) that break down gastrointestinal mucins and use them as a carbon source. Many types of *mucinolytic* bacteria break down mucin in the digestive tract and use it as a carbon source [19,50]. Primarily, mucolytic bacteria are found in healthy humans and animals. Among them, *B. acidifaciens* prevents obesity and improves insulin sensitivity in mice [51]. The next most abundant bacteria were the *Firmicutes-Clostridia-Lachnospiraceae* group (*Lachnospiraceae*, L. NK4A136, *L. bacterium*, ASF356, *Anaerostipes*) [49] and *Firmicutes Oscillospirales* order (*Ruminococcaceae*, *Anaerotruncus*, *Oscillospiraceae*, *Colidextribacter*, *Clostridium* sp.). Bacterial species belonging to the *Oscillospirales* family were positively associated with increased serum antioxidant enzyme activity, such as superoxide dismutase, glutathione peroxidase (GSH-Px), and a suggested catalase [52]. However, by 64 weeks, *Lactobacillus* (*Firmicutes-Bacilli-Lactobacillales-Lactobacillaceae*) had replaced *Bacteroidales* as the main constituent bacteria. Additionally, *Lachnoclostridium* and *Roseburia*, which belong to the *Lachnospiraceae* family, and *Adlercreutzia*, *Adlercreutzia* mucosicola, and *Gordonibacteria*, belonging to the *Eggerthellaceae* family of the Actinobacteria phylum, were significantly increased. These bacterial species have been shown to play a role in converting bioactive plant secondary compounds, such as daidzein from soybeans and trans-resveratrol from grapevines [53]. Similarly, from 53 to 64 weeks, the number of gut bacteria exhibiting characteristics of the AL diet decreased by approximately 50%, indicating that the microbiota was completely different.

For the ALA group, the diversity of bacterial flora slowly increased, even up to 64 weeks of age. In particular, the mucolytic bacterial species belonging to the *Bacteroidales* order (*Muribaculaceae*, *B. acidifaciens*, *B. massiliensis*, *Alloprevotella*, *Muribaculum*, *Bacteroides*, *Odoribacter*, and *Parabacteroides distasonis*) increased significantly. These bacteria coexisted with the *Firmicutes-Clostridia-Lachnospiraceae* group [49] (*L. bacterium*, ASF356, *Anaerostipes*, *E. ventriosum* group) and the *Oscillospirales* group [52] (*Ruminoccaceae-Ruminoccaceae-Anaerotruncus*, *Oscillospiraceae-Colidextribacter*) to form a well-balanced environment consisting of the gut microbiota. Moreover, *Turicibacter* [44], an SCFA-producing bacterium that induces the production of T reg cells; the *Clostridia* vadinBB60 group [54]; and *Bilophila* [55], which belongs to the phylum *Desulfobacteria*, were present in the intestines of the ALA group.

Figure 2B shows the time-dependent changes in the average body weight of AC, AL, ALA, and WC mice from 12 to 53 or 12 to 64 weeks. The body weights of AC and WC mice fed only with commercial diets increased with the number of weeks of rearing. The body weight of AC mice decreased rapidly at 53 weeks, when the diversity of the intestinal microflora decreased (Figure 2A), whereas the body weight of WC mice gradually increased until 64 weeks, after which it decreased along with the decrease in the diversity of the intestinal microflora.

In contrast, the body weight of limonoid-fed AL mice and limonoids + L-arginine-fed ALA mice increased with the increase in number of weeks of rearing, but this process was very slow compared with the AC and WC mice. AL mice also began to lose weight at week 64 (Figure 2A), when the diversity of the gut microbiota decreased; ALA mice, with the richest gut microbiota diversity at week 64, also had the highest body weight. Figure 2C presents the mean weight of mice at 12–53 weeks (AC) or 12–64 weeks (AL, ALA, WC), which was 33.7 g ± 2.87 (mean ± SD), with the AC group being the heaviest, followed by the WC at 32.6 g ± 3.73, AL at 29.4 g ± 2.19 (*p* < 0.001), and ALA groups at 28.7 g ± 4.93 (*p* < 0.001). Thus, the average body weight of the AC mice was 20% higher than that of the ALA mice. From among the AC, AL, ALA, and WC mice, the average dietary intake of the AC mice was always higher during the 12–53 weeks of rearing Figure 2D, and the average intake of food during the entire rearing period was significantly higher (4.67 g ± 0.331 g/mouse day^−1^) (Figure 2E, *p* < 0.001). The mean food intake of the AL, ALA, and WC mice was not significantly different during the 12–64 weeks of rearing (Figure 2D), and the mean food intake during the whole rearing period was 3.22 ± 0.305 g/mouse day^−1^, 3.16 ± 0.312 g/mouse day^−1^, and 3.36 ± 0.170 g/mouse day^−1^, respectively. The average intake of the AC group was 32.3% higher than that of ALA, which had the lowest average food intake, indicating an unusual appetite.

These results suggest that the reason for the increase in body weight of WC mice compared with that of the AL and ALA mice is unlikely to be the amount of food they were fed and the suppression of body weight gain in AL and ALA mice compared with that in AC and WC mice was due to the dietary intake of limonoids and L-arginine.

**Figure 3:**
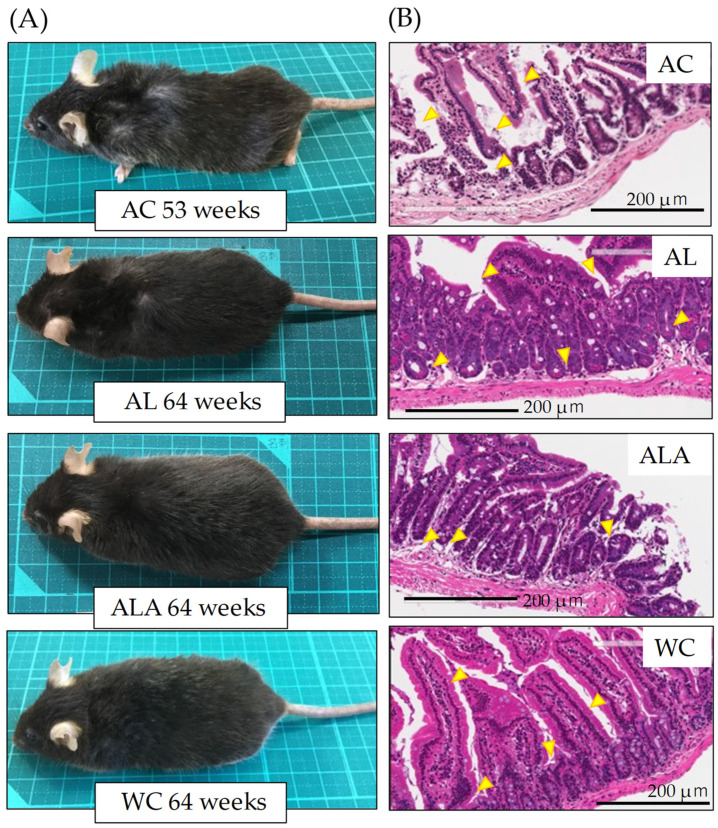
Observations of the appearance (**A**), small intestine tissues (**B**), in mice at 53 weeks of age (AC) or 64 weeks of age (AL, ALA, and WC). (**B**) Histological observations by H&E staining of small intestine tissues. Arrows indicate the destruction of the jejunal epithelium. Original magnification: 200×. Scale bar = 200 μm. AC, AL, or ALA mice were fed a normal diet, containing limonoids alone, and L-arginine + limonoids, respectively. WT mice (WC) were fed a normal diet.

Figure 3A,B presents the appearance of the AC (53 weeks), ALA, AL, and WC mice (64 weeks) and H&E-stained small intestinal tissue sections, respectively. Figure 3A shows that AC mice (53 weeks) showed obvious alopecia and whitening of body hair due to scratching and wounding caused by intense itching and mutual aggression. AL mice (64 weeks) showed a slight alopecia of body hair due to scratching caused by itching. Conversely, ALA (64 weeks) and WC (64 weeks) mice did not demonstrate aggression throughout the rearing period against other mice and were calm. In WC mice, bleaching of the tips of the body hair was observed.

H&E staining of the tissue sections of the small intestine showed histopathologically significant severe small intestinal mucosal injury in AC mice (53 weeks), disrupting the epithelial cell structure and causing massive infiltration of lymphocytes into the mucosa and submucosa. In AL mice, the surface of the small intestinal mucosa was markedly injured compared with ALA and WC mice, and the injury partially extended to the deep mucosa (Figure 3B, arrows in AC and AL). However, ALA and WC mice also showed separation of the epithelium from the mucosal muscle plate, irregular branching of the glandular structures, and elongation of the crypt (Figure 3B ALA, WC arrows), suggesting that there was inflammation in the small intestine at 64 weeks (pathology core pictures in the Japanese Society of Pathology (as outlined in https://Pathology.or.jp/ accessed on 15 August 2021)).

### 3.2. Observation of Bacterial Translocation in AD Mouse Model

**Figure 4:**
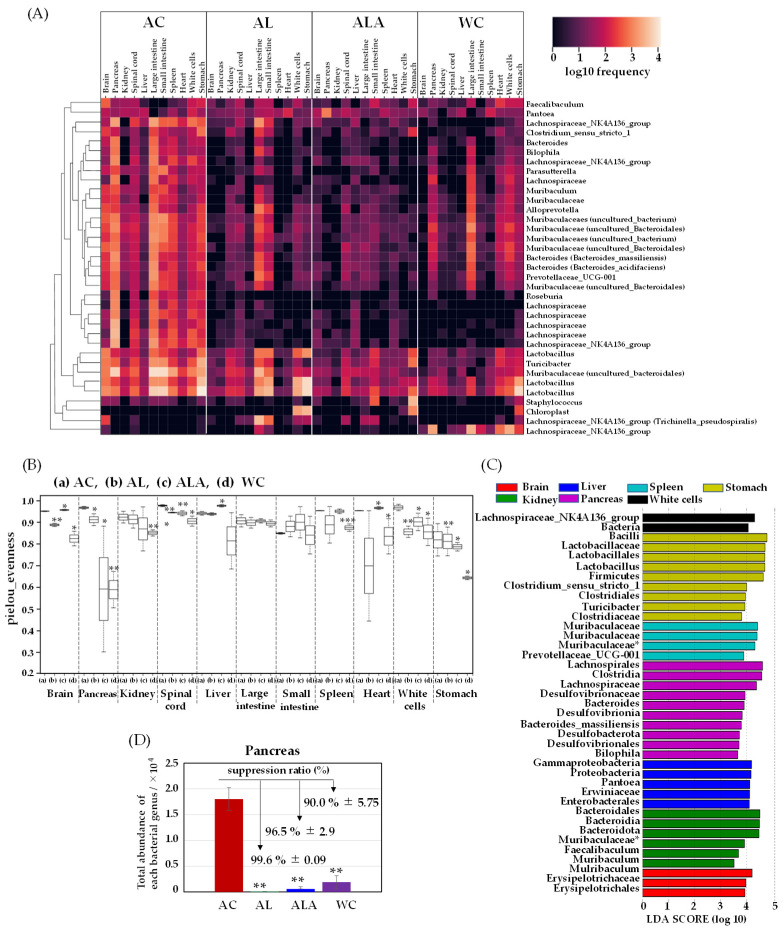
A clustering analysis of bacteria in the mouse tissues (brain, pancreas, kidney, spinal cord, liver, large intestine, small intestine, spleen, heart, white cells, and stomach). Heatmap (**A**) and alpha diversity (**B**) of genus taxonomic categories identified by genomic analysis of 16S rRNA in tissues of mice aged 53 weeks (AC) or 64 weeks (AL, ALA, and WC mice). Data are plotted in comparison with the Alzheimer’s disease control (AC) mice for clarity; all tissues: *n* = 3/each, * *p* ≤ 0.05, ** *p* ≤ 0.01, *** *p* ≤ 0.001. (**C**) Histogram of the LDA scores (log10) > 3, *p* < 0.05 computed for features differentially abundant in the tissues of AC mice. (**D**) Total bacterial abundance ratio of genus taxonomic categories identified by genomic analysis of 16S rRNA in the pancreatic tissue of AD model mice aged 53 weeks (AC) or 64 weeks (AL, ALA, and WC). AD mice were fed limonoids alone (AL), L-arginine + limonoids (ALA), or the normal diet alone (AC). Wild-type mice were fed the normal diet alone (WC). * Bacteria with species-level taxonomic categories.

Approximately 1000 species and 100 trillion bacteria are present in the human gastrointestinal tract [56]. Therefore, we speculated about how the bacterial flora existing in the intestines of AC disappeared. To this end, we examined the translocation of gut microbiota to the brain, pancreas, kidney, spinal cord, liver, large intestine, small intestine, spleen, heart, white cells, and stomach tissues of 53-week-old AC, 64-week-old ALA, AL, and WC mice. We measured the presence of bacteria derived from enteric bacteria in the brain, pancreas, kidney, spinal cord, liver, large intestine, small intestine, spleen, heart, white cells, and stomach tissues of ALA, AL, and WC mice. Surviving mice were dissected under anesthesia, and all organs were immediately frozen in liquid nitrogen at −80 °C. Figure 4 shows the results of the correlation analysis (Heatmap) between the number of bacteria present in each tissue of AD model mice by next-generation sequencing using 16s rRNA.

A large amount of bacteria were found in all organs of AC mice, including the brain, pancreas, kidney, spinal cord, liver, large intestine, small intestine, spleen, heart, white cells, and stomach. Invasion of the bacteria in the pancreas was the most severe, indicating the presence of most of the bacterial species in this organ. Although the number of tissue bacteria in ALA and AL was negligible, the presence of bacteria was confirmed. Particularly, *Firmicutes-Erysipelotrichaceae-Turicibacter*, *Firmicutes-Lactobacillaceae-Lactobachillus*, and *Bacteroidota-Muribaculaceae* were found in all tissues. The bacterial abundance in each organ was higher in AC mice, but the diversity of bacteria in each tissue was also higher in most AC tissues (Figure 4B). We found that the bacterial diversity in WC tissues was much lower than that in AC tissues of the brain, pancreas, kidney, spinal cord, heart, white cells, and stomach (** *p* ≤ 0.01 or *** *p* ≤ 0.001 for AC). Bacterial diversity was also significantly different (** *p* ≤ 0.01) between ALA and WC mice.

The microbiota communities in the AC mice tissues were diverse compared with those in the other mice (AL, ALA, and WC). We then performed LEfSe analysis to compare the estimated microbiotas in the AC mice tissues. A histogram of the LDA scores was computed for features that showed differential abundance between the AC mice and other mice (Figure 4C). The LDA scores indicated the relative abundances of *Bacteroidales-Mulribaculum* and *Erysipelotrichaceae* in the brain and *Proteobacteria-Gammaproteobacteria-Enterobacterales Pantoea*, *Muribaculaceae uncultured Bacteroidales*, and *Firmicutes-Bacilli-Faecalibaculum* in the kidney (LDA score (log10) > 3, *p*< 0.05). *Enterobacterales* in the liver, *Pantoea* in the spleen, and *Muribaculaceae-Muribaculaceae* were also identified. In the spinal cord, species with the same tendency as those in the brain were present. In the stomach, *Firmicutes-Lactobacillus*, *Firmicutes-Clostridium sensu stricto 1*, *Firmicutes Erysipelotrichaceae*, and *Turicibacter* and in the white cells, *Firmicutes-Clostridium sensu stricto 1*, *Firmicutes Erysipelotrichaceae*, and *Turicibacter* were analyzed as biomarkers. In the white cells, Firmicutes-Clostridia-Lachnospiraceae-NK4A136 group and in the pancreas, Bilophila, which belongs to *Desulfobacterota* and produces hydrogen sulfide that promotes inflammation in the intestine, were observed. *Bacteroides massiliensis* and *Firmicutes-Clostridia-Lachnospiraceae*, which produce hydrogen sulfide and promote inflammation in the intestine, were analyzed as characteristic bacteria and showed trends different from those of other tissues.

Figure 4D shows the total bacterial expression in the pancreas where the bacterial translocation (BT) [57] was the most abundant. In the pancreatic tissue of the AC mice, 275-fold higher bacterial expression compared with that in the AL mice, 31-fold higher bacterial expression compared with that in the ALA mice, and 9.4-fold higher compared with that in the WC mice was observed. Suppression ratio of bacterial expression was achieved up to 99.6% ± 0.09 and 96.5% ± 2.9 in the AL and ALA groups, respectively. Bacterial expression was more negligible in AL and ALA mice than in the pancreas of WC mice (90.0% ± 5.75). It has been reported that 60% of the patients with acute human pancreatitis have disrupted intestinal barrier [58,59]. Analysis of the intestinal microbiota of human patients with severe acute pancreatitis reported a high prevalence of Enterobacteriaceae and Enterococcus and a low prevalence of *Bifidobacteria* [60]. The bacterial composition in human patients with acute pancreatitis was like that observed in the AL mice but significantly differed from that in the WC group. In contrast, in AC mice, significant exudation of *Enterobacterales Pantoea* was observed not only in the pancreas but also in the kidney and liver in large amounts (Figure 4A,C).

### 3.3. Histopathological Analysis of Pancreas, Liver, and Brain Tissue

**Figure 5:**
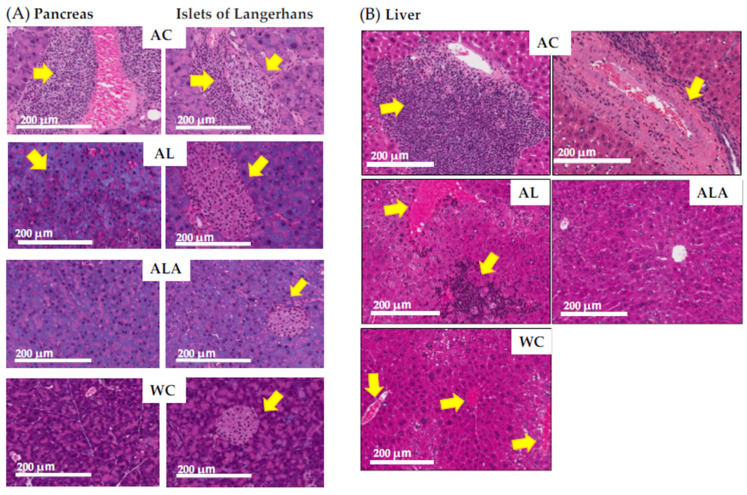
Histological observation of small intestinal tissue by H&E staining. Photomicrographs of pancreas (**A**) and liver (**B**) are representative of tissue cross sections from the AC, AL, ALA, and WC mice. Arrows indicate tissue lesions and study sites. Magnification in the original paper is 200×. Scale bar = 200 μm.

The H&E staining results of the pancreas showed the destruction of pancreatic ductal epithelial cells and infiltration of condensed proteins and eosinophils in the pancreatic duct of AC mice (Figure 5A), and tumor growth was observed in the surrounding area. Tumors were also expressed in the tissues surrounding the enlarged islets of Langerhans, indicating severe and devastating inflammation of the pancreatic tissues.

In AL mice, eosinophilic infiltration, and hypertrophy of the pancreatic islets of Langerhans and lipidification of the cells were observed (see overview in pathology core pictures in the Japanese Society of Pathology, https://Pathology.or.jp/ accessed on 15 August 2021). The pancreatic islets of Langerhans and acinar cells in ALA and WC mice were similar in shape.

Figure 5B shows sections of liver of the AC mice with marked portal mononuclear cell infiltration by inflammatory cells, edematous fibrous tissue, hyalinization of hepatic arterioles, and congestion of portal vein, which displays the severely affected hepatocytes with pyknotic nuclei and fibrosis in the portal area. The liver of the ALA mice was presented with normal hepatocytes and vesicular nuclei arranged in branching cords around the central vein with the normal portal area, central vein, and normal ducts. The liver of the AL mice showed increased eosinophil infiltration and infiltration by inflammatory cells, with steatosis in some hepatic cells. The liver of the WC mice indicated eosinophilic infiltration.

**Figure 6:**
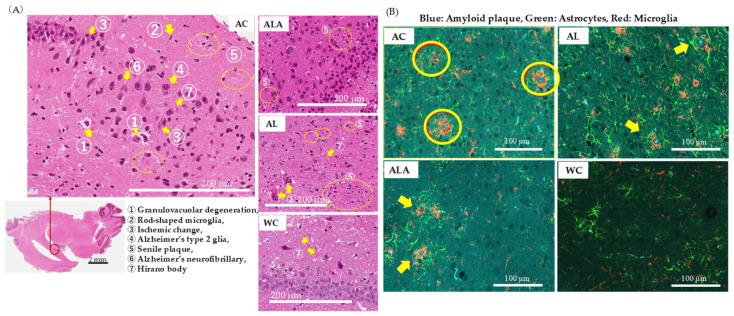
Histopathological analysis of brain tissues of 53-week-old (AC) or 64-week-old (AL, ALA, and WC) mice. (**A**) H&E staining of the regions around the hippocampus (original magnification: 200×, scale bar = 200 μm) and (**B**) immune triple fluorescence staining of regions around the hippocampus (original magnification: 400×, scale bar = 100 μm) of AC, ALA, AL, and WC mice. The sections show senile plaques formed with the amyloid β storage cells. (**B**) Triple immunofluorescence staining images of hippocampal samples localized Amylo-Glo-stained amyloid plaques (blue), GFAP-positive astrocytes (green), and IbA 1-positive microglia (red) on the same section. In the AC mice, hypertrophied astrocytes and activated microglia were upregulated around the amyloid plaques, forming senile plaques.

Figure 6A shows the results of the histopathological analysis of the hippocampal specimens from AD mice as outlined by the Tokyo Metropolitan Institute of Medical Science (https://igakuken.or.jp/ accessed 20 August 2021).

In AC mice, age-related changes in hippocampal neurons, as well as the presence of granulocytosis, Hirano bodies, Alzheimer’s neurofibrillary changes, ischemic changes, and senile plaques, all of which are common in Alzheimer’s disease, were observed. In addition, rod-shaped reactive microglia with club-shaped nuclei were observed showing typical inflammatory and autoimmune lesions. Alzheimer’s type 2 glia with greatly enlarged astrocyte nuclei were also observed, suggesting the possibility of hepatic encephalopathy due to severe liver damage. In ALA, such characteristic histopathological degenerations reduced, and the degeneration of the granular layer was also minimized, confirming the difference between these and the hippocampal specimens of WC mice showing physiological aging. However, AL mice showed increased granulocytopenic degeneration and senile plaques compared with the ALA and WC, indicating enhanced characteristics of neurodegenerative diseases. The astrocytes in hippocampus of the AL mice were activated and widespread aggregation of microglia with amyloid plaques at the center was evident. In ALA mice, senile plaque formation could be observed in some areas, but the expressions of astrocytes and microglia were not enhanced and appeared like those in WC mice.

These results (Figure 5 and Figure 6) confirmed that the pancreas, liver, and brain of AC mice with dysbiosis in the gut microbiota were in a severe state of tissue and hippocampal neurons destruction and the tissue and hippocampal neurons of the AL group was also damaged. The intestinal microbiota and pancreatic and liver tissue and hippocampal neurons homeostasis was maintained to a better extent in the ALA mice than in the WC mice.

### 3.4. Metabolomic Analysis of Mouse Liver and Feces

**Figure 7:**
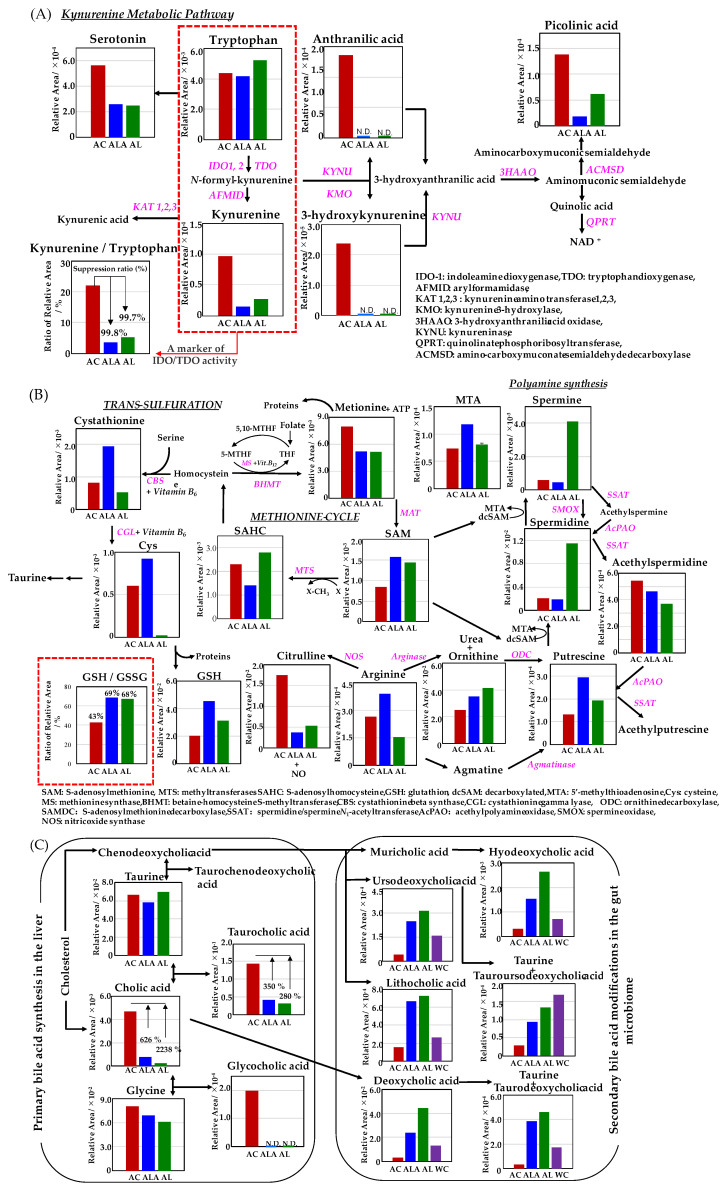

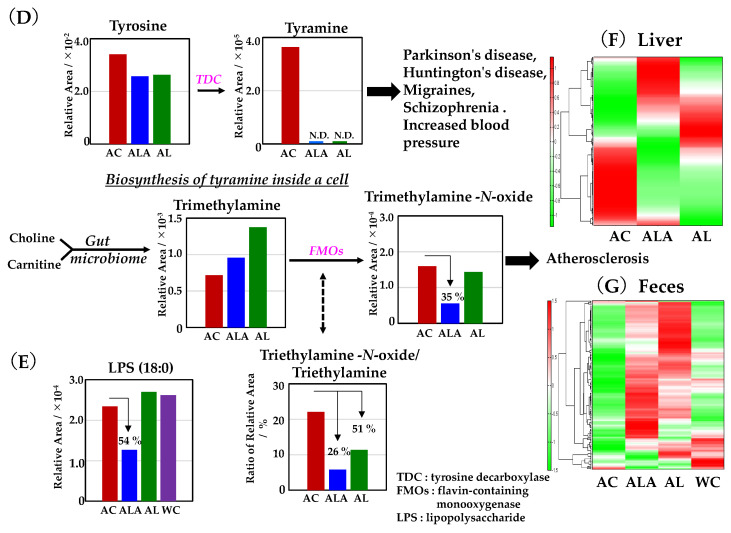
Metabolite analyses results of mouse liver and feces using CE- and LC-TOFMS. (**A**) Simplified schematics of the kynurenine pathway. (**B**) Simplified schematic of the metabolic pathway related to the methionine cycle, urea cycle, and polyamine metabolism compounds. (**C**) Metabolic simplified schematic pathways of primary and secondary bile acids. The production process of tyramine and trimethylamine-*N*-oxide (**D**), and lipopolysaccharide (LPS) (**E**). Heatmap showing hierarchical clustering analysis of metabolomic results of the liver (**F**) and feces (**G**). AD mice were fed diets containing L-arginine + limonoids (ALA), limonoids alone (AL), or a normal diet alone (AC).

Metabolomic analysis was performed on the liver of AC, ALA, AL mice and the feces of AC, ALA, AL, and WC mice. The kynurenine metabolic pathway is shown in Figure 7A; the methionine and urea cycles and metabolite pathways related to polyamine metabolism are shown in Figure 7B; and the overview of the bile acid metabolic pathway is shown in Figure 7C. Other metabolites believed to be involved in AD are shown in Figure 7D,E, and a heatmap showing hierarchical clustering analysis of the AD mice liver and feces metabolomic results are shown in Figure 7F,G.

In the kynurenine pathway (Figure 7A), all major metabolites metabolized from tryptophan were strongly upregulated in AC mice. In human patients with AD and those with mild cognitive impairment, the kynurenine/tryptophan ratio, which has been reported to be about twice of that found in healthy subjects [61,62], was approximately 22% for AC, 3.6% for ALA, and 5.2% for AL. In our studies, suppression of the kynurenine/tryptophan ratio was achieved up to 99.8% and 99.7% in ALA and AL mice, respectively. In addition, 3-hydroxykynurenine, which is listed as a toxic metabolite that may exhibit neurotoxicity and induce oxidative damage and cell death in brain neurons [63], and anthranilic acid, for which elevated plasma levels and a risk associated with dementia have been reported [64], were identified only in the AC mice.

Homocysteine, an intermediate metabolite of the methionine cycle, is converted by the trans-sulfuration pathway to cystathionine and cysteine, one of the constituent amino acids of glutathione (Figure 7B), which is essential for detoxification and antioxidation in living organisms [65].

ALA upregulated both cystathionine and cysteine produced by the trans-sulfuration pathway, and GSH production showed ALA > AL > AC, with ALA mice producing about twice as much GSH as AC mice. Glutathione is an intracellular tripeptide with an SH group, which are of the following two types: reduced GSH and oxidized glutathione-S-S-glutathione (GSSG). Reduced GSH is converted to oxidized GSSG to detoxify hydrogen peroxide (H_2_O_2_) generated in vivo, and GSSG is regenerated to GSH by glutathione reductase. In healthy cells and tissues, reduced GSH accounts for most of the GSH; therefore, changes in the GSH/GSSG ratio can predict the degree of inhibition of intracellular oxidative stress [66]. The % values of GSH/GSSH were compared, and the ratio of GSH production was approximately 69% in ALA, 68% in AL, and 43% in AC.

Thus, the intracellular oxidative stress was more suppressed in ALA and AL mice than in AC mice. Furthermore, ALA increased the metabolism of S-adenosylmethionine (SAM) [66], an essential metabolite that regulates major metabolic pathways, including methylation and polyamine synthesis, as well as arginine, putrescine, and 5′-methylthioadenosine (MTA) in the arginase–polyamine synthetic pathway in the methionine cycle. The AL mice showed the presence of more metabolites of ornithine, spermidine, and spermine in the arginase–polyamine synthetic pathway, than AC and ALA mice. In the AC mice, in contrast, the nitric oxide synthase pathway, which produces nitric oxide (NO) from arginine, and the spermidine/spermine-*N*_1_-acetyltransferase and spermine oxidase of the polyamine back conversion cascade demonstrated an increase in citrulline production and acetylspermidine in each pathway.

Bile acids produced by many mammals, including humans, are cholic acid (3α,7α,12α-trihydroxy-5β1cholanoic acid) and kenodeoxycholic acid (3α,7α-dihydroxy-5β-cholanoic acid). The bile acids produced in the liver are further conjugated with taurine or glycine and secreted into bile. The primary bile acids metabolized from cholesterol in the liver of mice are mainly accumulated in the gallbladder as taurine-conjugated bile acids and secreted into the duodenum through the bile ducts in response to dietary intake [67]. Figure 7C shows that in the liver of the AC, ALA, and AL mice, cholic acid, taurocholic acid (cholic acid conjugated with taurine), and glycocholic acid (cholic acid conjugated with glycine) were identified.

The number of primary bile acids metabolized was significantly higher in AC mice, with cholanoic acid production about 626% higher than that in ALA mice and 2238% higher than that in AL mice, and taurocholic acid production 350% higher than that in ALA mice and 280% higher than that in AL mice. Glycocholic acid was found only in the AC mice. The primary bile acids initially produced in the liver undergo various transformations by intestinal bacteria during enterohepatic circulation to produce secondary bile acids [68]. Secondary bile acids obtained from the feces of the AC (53 weeks) and the ALA, AL, and WC mice (64 weeks) in this study were metabolized from ursodeoxycholic acid, lithocholic acid, and cholic acid via chenodeoxycholic acid deoxycholic acid metabolized via chenodeoxycholic acid, and hyodeoxycholic acid and ursodeoxycholic acid induced by the chenodeoxycholic acid→muricholic acid metabolic pathway are obtained. The production of tauroursodeoxycholic acid and taurodeoxycholic acid conjugated with taurine was confirmed from deoxycholic acid. In the measurement of secondary bile acids, it was shown that the production of all secondary bile acids in the AC mice feces was slightly deficient. In the AL mice, all secondary bile acids production was the highest except for tauroursodeoxycholic acid, followed by that in the ALA mice; in the WT mice, taurodeoxycholic acid was produced most frequently.

In Figure 7D, tyramine metabolites converted from tyrosine by tyrosine-decarboxylase (TDC) were found only in AC mice. Choline and carnitine are metabolized by intestinal microflora to produce trimethylamine (TMA), which is further metabolized by flavin monooxygenases (FMOs) to trimethylamine-*N*-oxide (TMAO), an atherogenesis-promoting species [69,70]. The production of triethylamine-*N*-oxide was reduced to 35% in ALA mice compared with AC mice and 90% in AL mice. The triethylamine-*N*-oxide/triethylamine ratio is one of the accelerating indicators of atherosclerosis [70]. This ratio was suppressed by 26% in ALA mice and up to 51% in AL mice compared with that in AC mice.

Figure 7E illustrates lipopolysaccharide (LPS) production [71], which is one of the pathogen-associated molecular patterns (PAMPs) [72,73] on the outside of the cell wall of Gram-negative bacteria. The relative production of LPS in the feces of the AC, AL, and WC mice was similar; however, the feces of the ALA mice had a 54% lower production ratio compared with that of the AC mice (Figure 7E).

Furthermore, HeatMap (Figure 7F,G), based on the hierarchical clustering of the metabolite files of the liver and feces of AD mice, showed that the metabolic files (Figure 7F) differed greatly among AC, ALA, and AL mice and that the metabolism of ALA and AL mice was partially different, suggesting that dietary differences in L-arginine may have affected this. A comparison of metabolites in feces (Figure 7G) shows that the metabolites of AC mice are extremely biased; ALA and AL mice were rich in many metabolites and had different metabolic rates and that WC mice had more metabolites than AC mice but were biased compared with ALA and AL mice.

### 3.5. Production of Proinflammatory Cytokines and Chemokines in Serum of AD Model Mice and WT Mice

**Figure 8:**
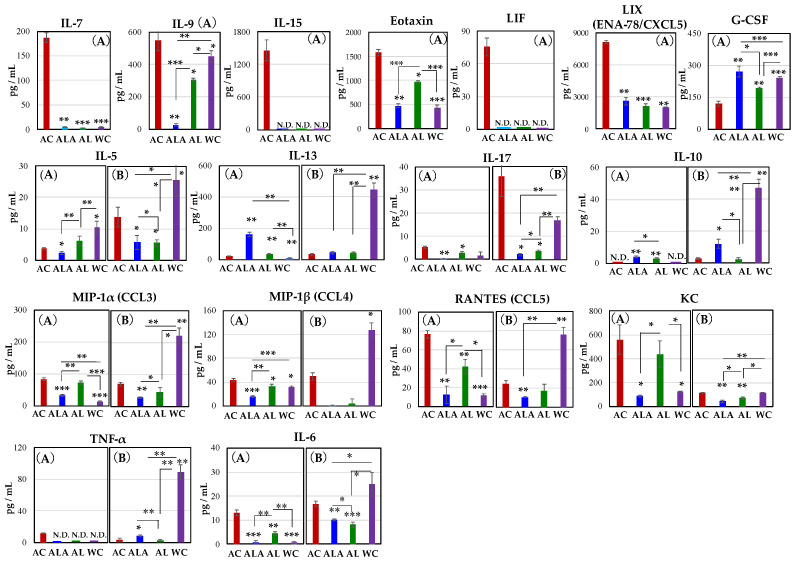
Alterations in the cytokine and chemokine levels in the serum of AC, ALA, AL, and WC mice at 30 (**A**) and 53 (AC only) to 64 weeks of age (**B**). Significant changes in the cytokine levels for 30-week-old AC mice (**A**): interleukin-7 (IL-7), IL-9, IL-15, IL-17, IL-6, eotaxin, leukemia inhibitory factor (LIF), LIX (ENA-78/CXCL5), MIP-1α (CCL3), MIP-1α (CCL4), RANTES (CCL5), KC, and TNFα. One-way analysis of variance and *t*-tests were performed for all statistical analyses (* *p* < 0.05, ** *p* < 0.01, *** *p* < 0.001).

We measured cytokines and chemokines in the serum of AC mice at 30 weeks of age when their gut microbiota was most diverse, and at 53 weeks (Figure 2A) when they were in dysbiosis (Figure 8), to compare the effects of their diets on the gut microbiota. The results of 30- and 53-week-old AC and 64-week-old ALA, AL, and WC mice are shown in Figure 8A,B, respectively.

As shown in Figure 8A at 30-weeks-old, IL-7, IL15, leukemia inhibitory factor (LIF), and TNF-α were found to be expressed only in AC mice. Increased expression of IL-7 is associated with exacerbated inflammation in the central nervous system and peripheral immune cells [74]. IL-7 is also a cytokine that indicates the acute phase of infection induced liver inflammation [75]. IL-15 is a mucosal immune-related cytokine, and its expression is induced in dendritic cells (DCs) upon LPS or IFN-γ stimulation and is elevated in epithelial cells upon infection [76,77]. IL-15 expression in aging mice and lymphatic endothelial cells of blood and peripheral lymphoid organs is significantly increased by LPS-induced inflammation [78]. Moreover, severe intestinal inflammation was reported to develop in mice with ulcerative colitis and high expression in mesenteric lymph nodes and the intrinsic layer of intestinal mucosa of the small intestine [79]. LIF is a member of the IL-6 family of cytokines that affects cell growth by inhibiting cell differentiation. Its effects on cell differentiation, bone metabolism, cachexia, neurogenesis, embryonic development, and inflammation have been previously reported [80,81].

The production of IL-9, eotaxin, LIX(ENA-78/CXCL5), IL-17, RANTES (CCL5), KC, and IL-6 was also significantly higher in AC mice than in ALA, AL, and WC mice. Especially, expression of IL-9 was approximately 21-fold higher in AC than in ALA mice. IL-9 is produced by activated Th2 cells, mast cells and eosinophils, and it enhances inflammatory responses [82]. In inflammation characterized by massive infiltration of eosinophils, IL-4 and IL-5 induce high levels of macrophage inflammatory protein-1α and KC and RANTES; however, IL-13 is the most potent inducer of eotaxin (CCL11) [83].

Eotaxin plays a key role in inflammatory response by mobilizing immune cells, such as eosinophils, basophils, and Th2 lymphocytes, and inflammatory state often involves abundant eosinophilic infiltration in the pancreas [84]. Eosinophils are multifaceted, multifunctional leukocytes involved in the initiation and propagation of numerous inflammatory responses, including those after parasitic, bacterial, and viral infections, tissue damage, tumor immunity, and allergic diseases [85]. Many factors influence eosinophil regulation that induces an increase in eosinophils [86]. KC chemokines are reportedly regulators that stimulate proinflammatory and profibrotic genes in the liver and induce potent liver inflammation [87]. Hence, the behavior of these cytokines and chemokines suggests that inflammation in the liver and pancreas is induced in AC and AL mice. Therefore, these cytokines were expressed mainly in the AC mice at 30 weeks of age and were suggested to be associated with exacerbated inflammation in the central nervous system, peripheral immune cells [74], and lamina propria of the intestine [76,77,78] and the acute phase during infection-induced liver inflammation [75].

Furthermore, RANTES, also known as CCL5, is a class of cytokines that promotes leukocyte migration and plays an essential role in various inflammatory phases [88]. In addition to cell migration, it activates T cells, monocytes, astrocytes, and neutrophils [89].

Moreover, it is expressed in neurons at the edge of a cerebral infarct in AD and is involved in angiogenesis and tissue repair [90]. The chemokine G-CSF concentration decreased in the following ALA > WC > AL > AC order. In the AC group, the expression of LIF [80,81] and G-CSF [91], which are involved in nerve survival, repair, and regeneration, was also induced until 30 weeks of age (Figure 2 and Figure 8). It has been shown that administration of G-CSF in stroke and Alzheimer’s mouse models can induce neurogenesis near the site of injury, leading to neurological and functional recovery [91,92,93]. The marked increase in LIF and G-CSF production in AC mice at 30 weeks of age suggests that there may be a competitive relationship between self-renewal and disease progression.

IL-5, IL-6, IL-17, IL-13, and MIP-1β increased in AC mice from 30 to 64 weeks. Notably, in 53-week-old AC mice, high expression of IL-17 was confirmed. Thus, the expressions of cytokines and chemokines in 53-week-old AC mice or 64-week-old ALA, AL, and WC (Figure 8B) were completely different compared with those in Figure 8A.

In Figure 8, cytokines, and chemokines other than G-CSF, IL-10, and IL-13 expressed in AC mice were suppressed in ALA or AL mice, and this effect was sustained for up to 64 weeks of rearing.

In Figure 8B, except IL-17 and the chemokine KC, the production levels of cytokines and chemokines of the WC mice at 64 weeks of age were significantly increased, compared with that in the AC, ALA, and AL mice, or the 30-week-old WC mice. The changes in cytokine and chemokine expression observed in WC mice are expected to be due to the effects of individual aging.

In the present study, the production of these proinflammatory cytokines and chemo-kines was significantly higher in 30-week-old AC mice, suggesting that neurodegeneration and systemic inflammation develop strongly in the brain, pancreas, liver, and intestinal epithelial mucosa, in AC mice. However, when AD mice were treated with L-arginine and limonoids, the production of these inflammatory cytokines and chemokines was suppressed.

## 4. Discussion

We have previously reported [23] that one of the major strategies for maintaining host health is the existence of a system that protects the bacterial flora from enterotoxemia, which increases the permeability of the epithelial cells of the small intestine.

In our study of 53-week-old Alzheimer’s disease mouse model (AC), we observed that IL-17 was expressed in intestinal epithelial cells approximately seven-fold higher than that at 30 weeks, and the gut microbiota of AC had a predominance of large amounts of *Staphylococcus* spp. [43], *Eubacterium ventriosum* group, and E. *coprostanoligenes* group [44] (Figure 2), and induced intestinal barrier dysfunction (Figure 3B), indicating complete dysbiosis. IL-17 is a proinflammatory cytokine that mobilizes monocytes and neutrophils to the site of infection, and the activation of IL-17 successively activates several downstream cytokines and chemokines, including IL-1, IL-6, IL-8, IL-21, TNF-β, and MCP-1 [94]. High expression of IL-17 induces the production of angiogenin-4 and phospholipase antibacterial proteins in the intestinal tissues and, consequently, the growth of Clostridium XIVa is specifically inhibited [95]. Therefore, in the AC mice, the intestine did not receive sufficient metabolites to induce an immune response, and accelerated intestinal inflammation is expected to occur from 30 weeks of age.

Furthermore, we observed a large amount of BT [57,96,97] to the pancreas, which then extended to the brain, kidney, liver, spinal cord, spleen, heart, and white cells in 53-week-old AC mice (Figure 4). In AC mice, the expression of eotaxin [85,86], RANTES [88,89], and KC [87], which induces severe inflammation in the pancreas and liver, was also high. In addition to chemokine receptors, RANTES can bind to (glycosaminoglycans) [98], and in blood vessels, RANTES is released from blood cells and aggregates on endothelial cells and acts as a “signpost” for immune cells, leading to the mobilization of blood monocytes and T cells to the lesions. In addition, microglia, which are immune cells in the brain that play an essential role in regulating the BBB, are attracted to blood vessels in response to RANTES released from vascular endothelial cells during systemic inflammation. After accumulation, they express CD68, a molecule involved in phagocytosis, and accumulate in astrocytes that constitute the (BBB), and then, they partially phagocytose the protrusions of astrocytes that constitute the (BBB) [99]. Therefore, when leakage is triggered by disruption of this structure, abnormalities in BBB function occur [100]. It has been reported that exposure of microglial cells to bacterial lipopolysaccharide promotes the activation of cytokines, such as the IL-7 promoter [74]. In fact, in the brains of AC mice, the expression of hypertrophied astrocytes and activated microglia around the amyloid squad was increased, thus confirming the characteristic lesions of AD (Figure 6).

There is no doubt that one of the entry points involved in BT involves inflammation of the pancreas. Kurashima et al. found that a glycoprotein called glycoprotein 2 (GP2), which is secreted in large amounts by the pancreas, captures the surface of the intestinal bacteria (nematocysts) and suppresses their translocation into tissues [101]. It has been reported that when the secretion of this pancreatic protein is impaired by pancreatic inflammation, the transfer of intestinal bacteria into the tissues and blood is more likely to occur, leading to the severity of inflammatory bowel disease [101].

In summary, we can predict that tissue destruction of the host digestive organs by inflammation is the first cause for BT of bacteria present in the gut microbiota, which was identified in tissues throughout the body of the AD mice.

The tumors that developed in the pancreas of AD mice in this experiment and the destruction of the islets of Langerhans completely explained the presence of inflammation (Figure 5). Furthermore, liver metabolites of tyramine were found only in AC mice. Tyra-mine is an indirect sympathomimetic; when the body produces too much tyramine, it releases the neurotransmitter norepinephrine (NE) stored in the endoplasmic reticulum. This causes a marked increase in axonal NE levels and triggers the carrier-mediated transport of NE from nerve endings [102]. Excess NE has been shown to activate the sympathetic innervation of pancreatic beta cells and inhibit insulin secretion [103,104]. Normally, the enzyme monoamine oxidase (MAO), present in the outer mitochondrial membrane throughout the brain, liver, and other tissues, deaminates tyramine and produces hydrogen peroxide as a byproduct [105]. The presence of reduced glutathione (GSH) in the mitochondria helps to overcome the toxicity of peroxides. As the GSH/GSSG (oxidized glutathione) in AC mice obtained in this study was as low as 43% and tyramine has also been reported to have synergistic toxicity with amyloid-β42 [106], tyramine toxicity causing oxidative damage to cells in AD mice [107] is considered to be strong in AC, suggesting that it causes mitochondrial dysfunction.

Tyramine binding to G-protein-coupled receptors in the synaptic cleft reduces the activity of serotonergic and dopaminergic receptors [107], and imbalance in tyramine levels has been reported to be associated with Parkinson’s disease, depression, schizophrenia, migraine, and elevated blood pressure [108,109,110,111]. Our results suggest that it is one of the reasons for the progression of pathology in AD. In addition, the disruption of the gut microbiota in AC mice also disrupted liver function.

One of the important metabolites produced by gut bacteria is bile acids, an endogenous molecule synthesized from cholesterol in the liver [112]. These molecules can activate receptors in the gut, liver, and periphery to regulate several host processes, including metabolic processes [112]. Primary bile acids metabolized in the liver undergo conjugation by glycine or taurine and are transported to the liver in a less toxic form, where they are released into the duodenum after a meal. In the intestinal tract, intestinal bacteria induce the deconjugation and 7-alpha-dehydroxylation of primary bile acids to convert them to secondary bile acids. In our study, we found only in 30-week-old AC mice the strong expression of the inflammation-derived cytokine IL-15 [76,77]. In other words, in AC mice, LPS release from the outer membrane of Gram-negative intestinal bacteria occurred by 30 weeks of age, indicating that the liver was induced to produce inflammation. It is likely that high levels of secondary bile acids, deoxycholic acid (DCA), and lithocholic acid (LCA) in the liver were produced. DCA and LCA are known to induce inflammation in the liver, and the increased DCA, in particular, can induce cellular senescence and senescence-associated secretory process in hepatic stellate cells through enterohepatic circulation, senescence-associated secretory phenotype, thereby creating a microenvironment for hepatocellular carcinogenesis [113]. Excessive DCA metabolism is caused by the growth of Gram-positive bacteria such as Clostridium [114]. Gut microbiota at 30 weeks of AC was proliferated by the Clostridium group (Figure 2). This unbalanced bacterial ratio and the increase in secondary bile acids eventually led to the breakdown of the intestinal barrier, and lipopolysaccharide (LPS) and lipoteichoic acid (LTA) induced inflammatory signals through toll-like receptors in the liver.

The 53-week-old AC mice with dysbiosis of the intestinal microflora already showed a very high production ratio of primary bile acids and low levels of secondary bile acids in the liver (Figure 7). This suggests that the bile acids are cycling between the failing gut and the fibrotic liver until they can no longer be metabolized (Figure 3 and Figure 7C).

The pathological progression of AD also disrupted the function of the liver. In liver of AC mice, the high triethylamine-*N*-oxide/triethylamine ratio, which is one of the accelerating indicators of atherosclerosis [70], was found further.

Trimethylamine, a choline metabolite metabolized by intestinal bacteria, is hepatotoxic and involved in the development of nonalcoholic fatty liver disease (NAFLD)/nonalcoholic steatohepatitis (NASH). Choline promotes the transport of fat droplets in hepatic parenchymal cells and prevents excessive fat droplets in the liver. The choline metabolite trimethylamine is converted in the liver to TMAO and induces inflammation [115], and increased production of trimethylamine leads to choline deficiency and promotes fatty liver formation [116]. There is also a pathway from carnitine for metabolism to trimethylamine by intestinal bacteria. Like choline, it is metabolized to TMAO in the liver and has been reported to promote atherosclerosis [70,117].

The pathological progression of AD had also disrupted the function of the liver. In the kynurenine pathway, markedly abnormal tryptophan metabolism was identified. Kynurenine/tryptophan is known to correlate with the activity of indoleamine (2,3)-dioxygenase (IDO) [118]. IDO causes immunosuppression by degrading tryptophan in the tumor microenvironment and tumor-draining lymph nodes. Depletion of tryptophan and its toxic catabolites leads to inactivation of effector T cells and immunosuppression of dendritic cells. Inhibition of IDO is one of the targets for drug discovery and can slow tumor growth, enhance dendritic cell vaccination, and synergize with chemotherapy through immune-mediated mechanisms. ALA fed with L-arginine + limonoids and AL fed with limonoids alone suppressed 99.7–99.8% of the enhanced IDO activity in AC mice, as well as all other toxic metabolites of the kynurenine pathway [61,118].

Furthermore, a marked increase in abnormal AC metabolism was also observed in metabolic pathways related to the methionine cycle, urea cycle, and polyamine metabolism, while there were remarkable and impressive effects in ALA mice that were fed an L-arginine + limonoids diet. Dysregulation of S-adenosylmethionine (SAM) has been implicated in the development of cardiovascular disease [119], cancer [120], liver disease [121], and psychiatric disorders, such as depression and dementia [122].

In AD, oxidized species in brain tissues increase owing to the impaired activity of glutathione S-transferase, resulting in decreased levels of SAM and increased levels of its hydrolysis product S-adenosylhomocysteine (SAHC), which inhibits SAM utilization [123]. SAM can be synthesized in the cytoplasm of all cells, especially hepatocytes, and serves as a link in four major metabolic pathways, transmethylation, trans-sulfuration, and polyamine synthesis, and influences the regulation of autophagy [124,125].

Cysteine biosynthesis from SAM in trans-sulfuration is mainly active in the liver [126]. Cysteine is converted to a variety of sulfur-containing molecules such as glutathione (GSH), taurine, sulfate (SO_4_^2^^−^), and hydrogen sulfide (H_2_S). GSH protects many tissues from oxidative damage, and the depletion of GSH may induce autophagy. Thus, GSH redox homeostasis may be central to the maintenance of protein homeostasis achieved by autophagy regulation, and its maintenance is very important for inhibition of the pathogenesis of AD [127].

In the polyamine synthesis pathway, the enzyme adenosylmethionine decarboxylase converts decarboxylated SAM (dcSAM) from putrescine (Put), which is metabolized from L-arginine, agmatine, and ornithine, to spermidine (Spd) and spermine (Spm) [128]. dcSAM is metabolized to the neuroprotective factor MTA, which regenerates methionine [129]. In mice receiving dietary supplementation of L-arginine and limonoids diet, the levels of SAM were enhanced, and the production of MTA was also increased, balancing and controlling the pathway of citrulline + NO production from L-arginine (Figure 8B). Spd and Spm are essential polyamines involved in various cellular processes, including gene transcription and translation, DNA and RNA stabilization, signal transduction, and cell growth and proliferation [130,131]. They inhibit histone acetyltransferase activity and results in the upregulation of several autophagy-related genes (ATGs) [132].

The therapeutic effects of SAM in acute liver injury, hepatic fibrosis, and pancreatic cancer have also been demonstrated [133]. The hepatoprotective effects of SAM have been attributed to the inhibition of nuclear factor kappa B (NF-κB) translocation and the activation of nuclear factor erythroid 2-related factor (Nrf2), thereby suppressing oxidative stress and inflammatory responses in hepatocytes and pancreatic cells. This is thought to be due to the inhibition of oxidative stress and inflammatory reactions in hepatocytes and pancreatic cells by inhibiting the nuclear translocation of NF-κB and activating nuclear factor erythroid 2-related factor (Nrf2).

Excessive pools of Spm and Spd were observed in the polyamine metabolic pathway in the AL group; the Spm and Spd pools were enhanced by spermine oxidase (SMOX) in the polyamine back-transformation cascade. It has been reported that the chronic excessive production of Spm and Spd can cause liver injury and microglial activation [134] because the back-transformation reaction from Spm to Spd increases the level of free acrolein, which is strongly cytotoxic, as a byproduct via a SMOX-dependent mechanism [134]. However, dietary supplementation of limonoids to AD mice was more beneficial in terms of suppressing the AD pathology. However, the combined supplementation of L-arginine and limonoids did not result in excessive pooling of polyamines, but the reason for this is not clear so far.

In our study, we propose the possibility of inhibiting the pathological progression of AD in a multi-organ linkage, centered on the intestine, pancreas, liver, and brain. Our results suggest that the suppression of inflammation of the intestine, pancreas, liver, and brain in AD mice was achieved by the addition of L-arginine and limonoids to the diet of mice. We were not yet able to demonstrate a clear interaction between the gut microbiota, gut, pancreas, liver, and brain. However, a linkage between these organs certainly exists. Imai et al. [135] identified a neural network of liver-visceral nerve afferents-central nervous system-vagal centrifuge-pancreatic β-cells and reported the signal to initiate the increase in pancreatic β-cells was transmitted from the liver to the central nervous system through this network upon sensing obesity. Furthermore, Teratani et al. [136] reported that the liver functions as an information center that accurately accumulates and integrates the average information in the 8 m long human intestinal tract and transmits it to the brain without malfunction, and that there is a mechanism that transmits feedback from the brain to the intestine with appropriate commands to prevent excessive activation of intestinal immunity. In addition, it has been reported that there is a mechanism that transmits feedback from the brain to the gut with appropriate instructions for the gut situation to prevent the overactivation of intestinal immunity.

In the present study, L-arginine and limonoid supplementation in AD mice restored intracellular GSH level homeostasis, reduced oxidative stress, exerted anti-inflammatory effects by suppressing the secretion of proinflammatory cytokines, balanced the intestinal microflora, and promoted communication between the pancreas and liver to produce metabolites that promote host homeostasis. Our experimental results strongly supported a mechanism wherein the liver functions as an information center to prevent the overactivation of pancreatic and intestinal immunity and feeds back appropriate instructions from the brain to the gut–pancreas axis. Furthermore, it reaffirms that the maintenance of diversity in the gut microbiota has an immense impact on the maintenance of host life.

## 5. Conclusions

In this study, neurodegeneration of the brain, abnormalities in the gut microbiota, and BT to organs throughout the body, including the brain, were observed in AC mice; this induced functional failure of the gut, pancreas, liver, and brain tissue. Neurodegeneration was ameliorated and homeostasis of the host intestine, pancreas, liver, brain, and gut was improved by the dietary supplementation of a combination of L-arginine and limonoids; in addition, BT and aging were inhibited. These results suggest that L-arginine and yuzu seed limonoids may inhibit the progression of pathological symptoms in mouse models of AD.

## Data Availability

The data presented in this study are available in manuscript.
